# Exploring the effects of COVID-19 on motorcycle riding patterns and its importance

**DOI:** 10.3389/fpsyg.2023.994128

**Published:** 2023-01-30

**Authors:** Yukako Wada, Yoshifumi Bizen, Mitsuyuki Inaba

**Affiliations:** ^1^Faculty of Sport and Health Science, Ritsumeikan University, Kusatsu, Shiga, Japan; ^2^Faculty of Human Development, Kokugakuin University, Yokohama, Kanagawa, Japan; ^3^Institute of Advanced Research for Sport and Health Science, Ritsumeikan University, Kusatsu, Japan; ^4^College of Policy Science, Ritsumeikan University, Ibaraki, Osaka, Japan

**Keywords:** personal space, social distancing space, leisure activities, motorcycle riding, COVID-19

## Abstract

A motorcycle refers to a two-wheeled, personal mobility vehicle used for daily transportation and leisure activities. Leisure enables social interaction, and motorcycle riding could be an activity that facilitates social interactions and distancing. Therefore, grasping the importance of riding motorcycles during the pandemic—which involved social distancing and limited leisure activities—can be valuable. However, researchers have yet to examine its potential importance during the pandemic. Therefore, this study aimed to determine the importance of personal space and time spent with others in the context of motorcycle riding during the COVID-19 pandemic. We specifically explored the effects of COVID-19 on riding patterns and importance of riding motorcycle by examining whether these factors differed regarding changes in the frequency of motorcycle riding before and during the pandemic in daily and leisure-oriented transportation. Data were collected from 1,800 motorcycle users in Japan using a web-based survey conducted in November 2021. Respondents replied to questions concerning the importance of personal space and time spent with others attributed to motorcycle riding before and during the pandemic. Following the survey, we conducted a two-way repeated measures analysis of variance (two-factor ANOVA) and performed a simple main effect analysis using the SPSS syntax editor in case of interactions. The valid samples for motorcyclists with leisure motive (leisure-oriented users) and daily transportation motive (daily users) numbered *n* = 890 and *n* = 870, respectively (total *n* = 1,760, 95.5%). Each valid sample was divided into three groups based on the differences in motorcycle riding frequency before and during the pandemic: unchanged, increased frequency, and decreased frequency. The two-factor ANOVA results showed significant differences in the interaction effects for leisure-oriented and daily users regarding personal space and time spent with others. The mean value of the “increased frequency” group during the pandemic indicated significantly higher importance of personal space and time spent with others than the other groups. Motorcycle riding could enable daily transportation and leisure-oriented users to practice social distancing while simultaneously spending time with companions and alleviating loneliness and isolation during the pandemic.

## Introduction

The pandemic has globally restricted people from daily transportation and leisure activities for the past several years. COVID-19 has not allowed individuals to casually gather or spend time with others. Moreover, utilizing public transportation is recognized as having a higher risk of infection due to being in contact with many people, and people consequently preferred private vehicles over public transportation (Huang et al., 2020; Scorrano and Danielis, 2021). During the COVID-19 pandemic, people have been forced to stay at home instead of having face-to-face interactions; moreover, online communications, which maintain individual personal space, have become the mainstream way of connecting and spending time with others. Such social interactions have increased the sedentary lifestyles of people at home (Sivan, 2020). Although leisure time and activities offer good communication functions for promoting connectedness with others, the COVID-19 pandemic has made it difficult to have social interactions during leisure time (Norbury, 2021).

Motorcycles are widely used in daily life, especially in Asian countries, as a convenient means of transportation. In addition to daily transportation, many people enjoy riding motorcycles as a leisure activity. During COVID-19, the need to maintain social distancing to prevent infection has greatly restricted our daily lives. Under such circumstances, motorcycles are a one-person vehicle that have the advantage of allowing people to participate in activities while maintaining sufficient distance from others. Studies indicated that people changed their choice of daily transportation from public to private mode (Scorrano and Danielis, 2021), and outdoor leisure activities such as hiking, running, and cycling, which are enjoyable alone while ensuring personal space, became popular (van Leeuwen et al., 2020) as a result of the pandemic. The media reported that the COVID-19 pandemic led to a surging demand for motorcycle riding for daily transportation to practice social distancing (Kyodo News, 2022); this may also indicate that people chose motorcycles to ensure personal space and keep their distance from others. Additionally, according to the Japan Automobile Manufacturers Association, Inc [JAMA] (2022), the demand for motorcycles has increased because of the COVID-19 pandemic, and motorcycle riding may help avoid infection and the “Three Cs” (closed spaces, crowded places, and close-contact settings).

The COVID-19 pandemic has made people feel isolated and lonely (Sivan, 2020). Therefore, finding ways to practice social distancing while connecting with others could be essential during and after COVID-19 for new lifestyles and leisure activities or time. Has the impact of COVID-19 caused any change in motorcycle riding patterns and riders’ importance to motorcycle riding as the demand for motorcycles increases? It is assumed that the importance of riding motorcycles for riders enhanced for maintaining social distancing while spending time with others during the COVID-19 pandemic. However, researchers still need to examine the question. Thus, it is necessary to determine whether the riding patterns and riders’ importance to motorcycle riding have changed due to the COVID-19 pandemic.

## Literature review

### Maintaining personal space

We consider the distance between ourselves and others daily, either consciously or unconsciously. Moreover, everyone has a personal space—a distance that is comfortable for the individual. The concept of personal space is often used in social psychology and is defined as the emotional realm around an individual’s body. Personal space consists of physical and metaphorical aspects (Sommer, 1959). The meaning of personal space varies depending on the internal state, culture, and context. Therefore, personal space in leisure activities can also be considered a space detached from daily life (O’Brien et al., 2017). Many researchers have examined the relationship between personal space and human behavior.

For example, Gérin-Lajoie et al. (2008) examined changes in the personal space maintained around oneself during locomotion. They measured personal space in 10 adults while avoiding obstacles stationary in their path and found no relationship between walking speed and personal space. Motorcycles, the subject of this study, usually travel at speeds of 40 km/h or more, which is faster than walking. Therefore, examining personal space when riding a motorcycle will be necessary.

Welsch et al. (2019) examined the relationship between interpersonal distance and discomfort. People feel uncomfortable when their personal space is invaded. The researchers presented 15 different interpersonal distances ranging from 40 cm to 250 cm, and subjects rated their discomfort for each distance. The results revealed a correlation between personal space and discomfort. Regarding the relationship between gender and personal space, women get closer to other women when interacting (Hartnett et al., 1970). Moreover, Hecht et al. (2019) conducted experiments to measure personal space and found that a person’s personal space is circular with a radius of 1 m and that male-male pairs maintain more distance than female-female pairs or mixed-gender pairs. The Parietal-Frontal network of the human brain works to maintain interpersonal relationships and a specific “Comfort Zone” or personal space. Using fMRI experiments from a brain science perspective, Holt et al. (2014) found that the strength of the response between the dorsal intraparietal sulcus (DIPS) and ventral premotor cortex (PMv) is correlated with personal space and affects the preferred level of social activity.

The environment is a factor which determines human behavior, and research on how people utilize their environment to create social interactions has long been conducted with a focus on human behavior (Altman and Vinsel, 1977). In particular, as technologies such as virtual reality (VR) have evolved in recent years and the use of virtual environments has increased, there is a possibility that interpersonal distance in the real world has also changed. Kraut et al. (1998) found that increased Internet use causes a decrease in communication with family members and a smaller social circle, which is associated with increased depression and loneliness. While riding a motorcycle, one is basically in a state of maintaining distance from others. As social interaction and personal space change over time, it would be an interesting theme to examine the relationship between motorcycle riding and distance from others. Since the goal of this study is to examine the importance of social interactions with others in the context of motorcycle riding in the COVID-19 pandemic, we reviewed the following studies on social interaction with others.

### Social interactions regarding riding a motorcycle

Time spent with others and social interactions are crucial benefits of leisure (Sivan, 2020). The central concept of leisure refers to the free time in which an individual is neither working nor occupied by miscellaneous duties or obligations (Zijlstra and Sonnentag, 2006), and it is recognized as a pivotal time that includes activities that stimulate self-development and promote stress release (Liu et al., 2022). Furthermore, close relationships and leisure time with others promote subjective wellbeing (Newman et al., 2014; Hudson et al., 2020). The time spent with other people is critical as it could be related to individuals’ overall wellbeing.

Motorcycle riding as an outdoor leisure activity increases happiness in one’s life regardless of riding alone or in tandem (Kruger and Venter, 2020). There are two types of leisure—casual and serious. While casual leisure refers to relatively short-duration enjoyable activities that require neither special skills nor training, serious leisure entails pursuing special skills, knowledge, or experience, even as a hobby or at an amateur level (Stebbins, 1997). Motorcyclists have characteristics that include serious leisure, such as a strong sense of community and comradeship with other motorcyclists (Frash and Blose, 2019). Moreover, motorcyclists tend to have a “sense of belonging” or “sense of camaraderie” with other motorcyclists even when meeting for the first time or passing by each other on the road (Stokburger-Sauer, 2010; Walker, 2010; Frash and Blose, 2019). Therefore, riding a motorcycle might produce valuable opportunities to interact during the pandemic.

Scorrano and Danielis (2021) found that people had negative perceptions of riding public transportation, which led to a choice shift in choice from public to private vehicles during the pandemic; however, across motorized mobilities, such as a motorcycle and car, have low substitutability. Thus, motorcyclists’ frequency and importance of motorcycle riding may have changed before and during COVID-19. Understanding the riding frequency and importance of motorcyclists maintaining their personal space while spending time with others before and during the pandemic may lead to examining the utility of motorcycle riding in alleviating loneliness and isolation. Therefore, this study aimed to determine the importance of personal space and time spent with others in motorcycle riding during the COVID-19 pandemic. We specifically compared the importance of motorcycle riding based on changes in the frequency of motorcycle riding before and during the pandemic in both daily and leisure-oriented transportation.

People worldwide have been isolated and feel lonely due to the unexpected COVID-19 pandemic. We focused on the increased demand for outdoor leisure activities and purchasing motorcycles while people have been forced to change their lifestyles due to restrictions on daily transportation and leisure activities. Prior studies indicate that motorcycle is a convenient vehicle (Yamamoto, 2009; Crowther, 2011; Hanafi et al., 2019) and has the potential to enhance the quality of life (Newman et al., 2014; Hudson et al., 2020). Although a motorcycle is a personal vehicle with applications ranging from daily transportation to leisure activities, the importance of motorcycle riding has not been examined. Therefore, the present study might be valuable in demonstrating the usefulness of motorcycle riding for practicing social distancing and interaction in our lives. In particular, clarifying the growing importance of motorcycle riding in the post-pandemic lifestyle, outdoor leisure, and motorcycling-related academic studies could provide clues to new findings.

Practically, it remains unclear whether the demand for motorcycles during the pandemic was increased due to consumers’ need to maintain their personal space and leisure activities. Exploring whether COVID-19 affected motorcycle riding patterns and its importance and examining whether motorcycle riding contributes to maintaining personal space and social interaction would have implications for the wellbeing of people through motorcycle riding in their new lifestyle post-COVID-19.

## Materials and methods

We conducted a web-based survey in Japan using an Internet research company from November 18 to November 25, 2021. Respondents were registrants of the aforementioned internet research company, and they could participate the survey once. Data were collected from 1,800 Japanese motorcycle users aged 16 (18 for 401cc and above) to 69 who have purchased from Japanese motorcycle manufacturers (Honda, Kawasaki, Suzuki, and Yamaha). According to a survey (Japan Automobile Manufacturers Association, Inc [JAMA], 2022), motorcycle use can be categorized into two types: daily use (e.g., shopping and commuting to work) and leisure use (e.g., traveling on holidays). In our survey, we first asked about participants’ most recent motorcycle purchase experience, and those who responded with “yes” were asked about the manufacturers and purpose of using a motorcycle. The sample size was set and data were collected to avoid motorcycle manufacturers and displacement biases. As we expected that collecting data of participants with motorcycles under 250cc is easier compared to those with motorcycles over 251cc in Japan, we controlled to collect the same ratio of samples for both motorcycle displacements—under 250cc and over 251cc. Samples were asked about the purpose of purchasing the motorcycle using a multiple choice question. Valid samples were those who currently use motorcycles for daily transportation motives (daily users) and motorcyclists with leisure motives (leisure-oriented users).

Respondents answered questions regarding the importance of time spent with others and personal space due to motorcycle riding before and during the pandemic through Visual Analog Scales (VAS). The VAS is a psychometric rating scale similar to the Likert and Semantic differential scales and is recognized as a reliable scale for measuring pain within medical research studies (Bijur et al., 2001; Bielewicz et al., 2022). Respondents slid the point on a line rating from 0 to 100% in the VAS instead of choosing numbers in Likert scales. The VAS may take longer to answer than the Likert scale. However, Funke and Reips (2012) revealed no adverse effects of using the VAS, such as higher dropout, more non-response, or higher response times, on a Web-based survey. Moreover, the VAS can measure respondents’ perceived feelings objectively and is suitable for comparing respondents (Brazier et al., 2003). Thus, more precise data for measuring respondents’ feelings and recognition could be obtained by utilizing the VAS. Therefore, we used the VAS in this study and asked—regardless of the purpose for owning a motorcycle, daily transportation motives, or leisure-oriented purpose—whether motorcycle owners were able to maintain social distance and spend their time with others by riding a motorcycle during COVID-19. We specifically examined whether these factors differed based on changes in the frequency of motorcycle riding before and during the pandemic. Question items were, “How important is the personal space (individual space) provided by riding a motorcycle to you?” and “How important is the time you spend with your family and friends that you get from riding a motorcycle?” Respondents answered questions using the VAS, from (0%) “not important at all” to (100%) “very important” before and during the pandemic.

Following the survey, we conducted a two-way repeated measures analysis of variance (two-factor ANOVA) to compare the means of importance of riding a motorcycle regarding two factors: pandemic (i.e., before and during COVID-19) and motorcycle riding frequency. We examined a simple main effect analysis using the SPSS syntax editor in case of interactions *via* SPSS Version 27 (IBM Corp, 2020). Two-factor ANOVA includes two independent variables. Meanwhile, two-way repeated measures ANOVA is conducted when the independent variable includes comparisons of means between the same groups over time (e.g., pre and post). A simple main effect analysis will be required if interaction effects are present.

## Results

### Demographics of respondents

Approximately 90% of respondents were male, and their mean age was around 50 years of age. A previous research study mentioned that most motorcycling tourists were males over 40 years of age (Carter, 2017), and the Japan Automobile Manufacturers Association, Inc [JAMA] (2022) mentioned the mean age of the respondents as 54.2 years old in the survey. Therefore, the participants in this survey are representative of Japanese motorcyclists. About 60% of respondents were married with children. Concerning their occupation, about half of them were businesspeople, of which 5% were executives. The monthly discretionary amount was about 70,000 yen (US$500; 1US$ = 140 yen). Considering the monthly discretionary amount of the Japan Professional Football League stadium attendees is 36,100 yen (US$257.85; 1US$ = 140 yen) (Japan Professional Football League, 2020), the monthly discretionary amount of motorcyclists could be high.

The valid samples for motorcyclists with daily transportation motives (daily users) numbered *n* = 870 and leisure motives (leisure-oriented users) numbered *n* = 890, respectively (total *n* = 1,760, 95.5%). Valid samples were divided into three groups based on the differences in motorcycle riding frequency before and during the pandemic: (1) unchanged (daily users, *n* = 629; leisure-oriented users, *n* = 570), (2) increased frequency (daily users, *n* = 101; leisure-oriented users, *n* = 116), and (3) decreased frequency (daily users, *n* = 140; leisure-oriented users, *n* = 204).

As mentioned previously, this study included both leisure-oriented and daily motorcycle users and showed that approximately 90% of the respondents were male (daily users = 85.5%; leisure-oriented users = 93.4%), and their mean age was around 50 years. According to a report by Japan Automobile Manufacturers Association, Inc [JAMA] (2022), approximately 80% of motorcyclists in Japan ride solo, while only 20% ride in a group. Furthermore, the report revealed that more men than women ride solo (Japan Automobile Manufacturers Association, Inc [JAMA], 2022). Considering that about 90% of the participants in this survey were men, this result suggests that the majority of participants were riding solo. Regarding the engine displacements of motorcycles owned by the respondents, daily users owned smaller displacement engine motorcycles compared to leisure-oriented users; moreover, 40.5% of daily users owned under-50cc motorcycles, while half of the leisure-oriented users owned motorcycles over 401cc (see Table 1).

**TABLE 1:** Gender and age of respondents and their motorcycles’ engine displacement.

	Daily users					Leisure-oriented users	
	**Group**	***n***	**Male**	**Female**	***n***	**Male**	**Female**
Gender	(1) Unchanged	629	63.0%	9.3%	570	59.8%	4.3%
	(2) Increased	101	9.8%	1.9%	116	11.8%	1.2%
	(3) Decreased	140	12.7%	3.3%	204	21.8%	1.1%
	Total	870	85.5%	14.5%	890	93.4%	6.6%
		***n***	**Mean**	**SD**	***n***	**Mean**	**SD**
Age	(1) Unchanged	629	51.51	9.85	570	51.95	9.30
	(2) Increased	101	50.67	10.62	116	49.67	9.60
	(3) Decreased	140	51.16	10.62	204	52.23	8.60
	Total	870	51.36	10.06	890	51.71	9.21

		***n***	**Under 50cc**	**51–125cc**	**126–250cc**	**251–400cc**	**Over 401cc**	**Un-known**	***n***	**Under 50cc**	**51–125cc**	**126–250cc**	**251–400cc**	**Over 401cc**	**Un-known**
Engine displacements	(1) Unchanged	629	30.3%	22.6%	8.7%	5.3%	4.8%	0.4%	570	0.7%	5.2%	11.5%	12.5%	34.2%	0.1%
	(2) Increased	101	4.4%	3.4%	1.7%	1.7%	0.3%	0.0%	116	0.1%	1.1%	3.7%	2.8%	5.3%	0.0%
	(3) Decreased	140	5.7%	5.2%	1.8%	2.2%	0.9%	0.2%	204	0.0%	0.8%	4.6%	4.2%	13.4%	0.0%
	Total	870	40.5%	31.3%	12.3%	9.2%	6.1%	0.6%	890	0.8%	7.1%	19.8%	19.4%	52.8%	0.1%

### Results of two-factor ANOVA

**FIGURE 1:**
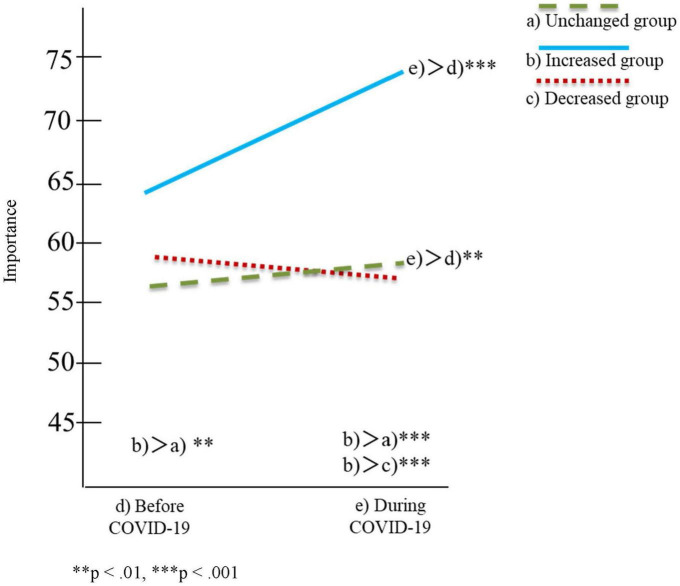
Personal space for daily users.

**FIGURE 2:**
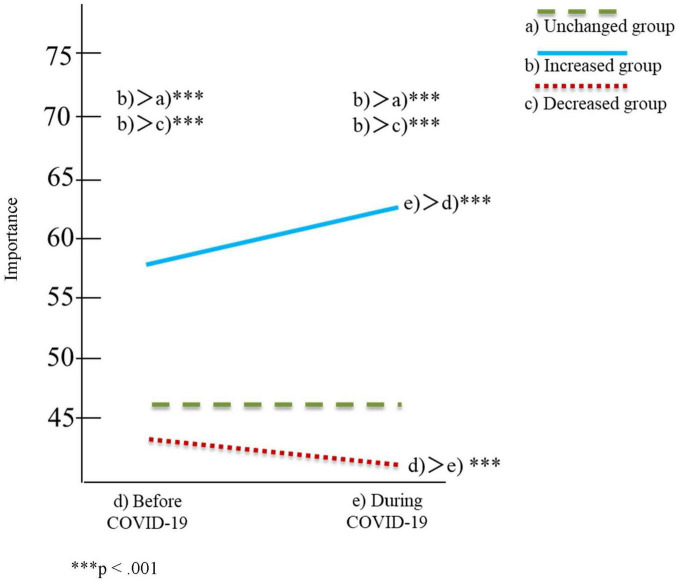
Time spent with others for daily users.

**FIGURE 3:**
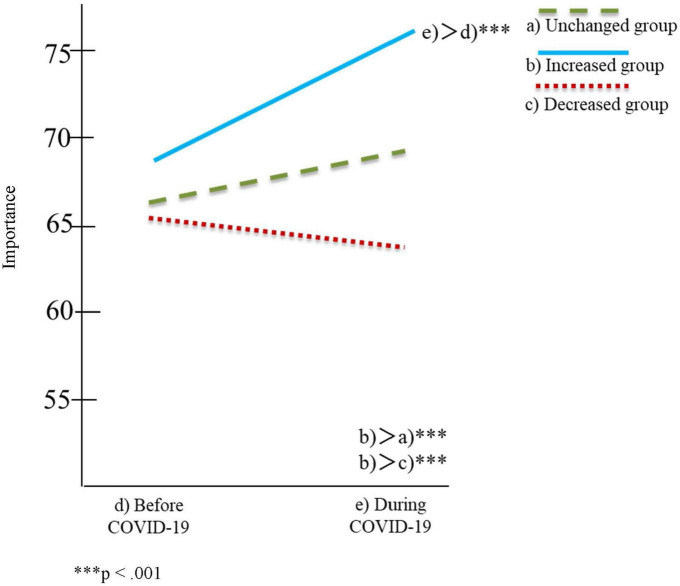
Personal space for leisure-oriented users.

**FIGURE 4:**
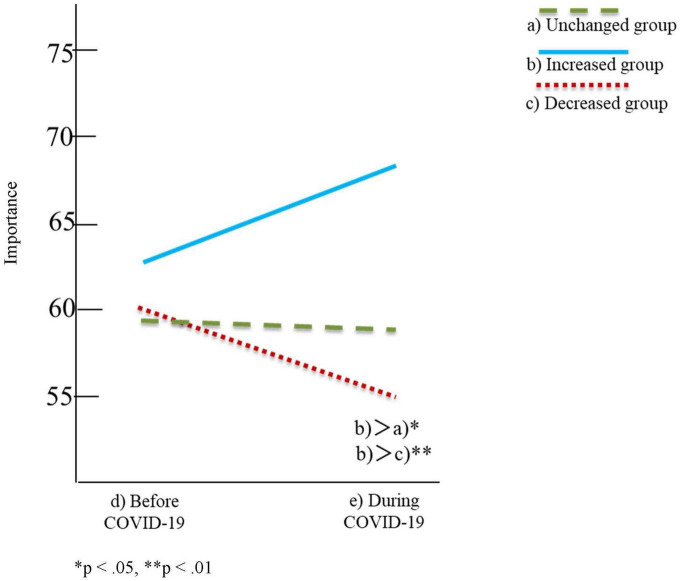
Time spent with others for leisure-oriented users.

Tables 2, 3 and Figures 1–4 show the results of the two-factor ANOVA and multiple comparisons for the importance of personal space and time spent with others for daily-oriented and leisure-oriented motorcycle users.

**TABLE 2:** Two-factor ANOVA and multiple comparisons for daily users.

**Personal space**	Interaction				Riding frequency		Before and during COVID-19
	***F*-value**	28.85***			13.55***		29.78***
	**Mean**	**SD**	**Mean**	**SD**	**Multiple comparisons**		**Multiple comparisons**
	**(d) Before**		**(e) During**		**(d) Before**	**(e) During**	
(a) Unchanged group	56.11	23.91	57.27	23.69	–	–	(e) > (d) **
(b) Increased group	64.70	22.01	74.36	19.71	(b) > (a) **	(b) > (a) *** (b) > (c)***	(e) > (d) ***
(c) Decreased group	58.54	5.93	56.68	25.95	–	–	–
**Time spent with others**	***F*-value**	18.43***			16.23***		10.15***
	**Mean**	**SD**	**Mean**	**SD**	**Multiple comparisons**		**Multiple comparisons**
	**(d) Before**		**(e) During**		**(d) Before**	**(e) During**	
(a) Unchanged group	46.81	25.80	46.98	25.95	–	–	–
(b) Increased group	57.57	25.41	63.47	26.66	(b) > (a) *** (b) > (c)***	(b) > (a) *** (b) > (c)***	(e) > (d) ***
(c) Decreased group	43.14	26.26	41.46	26.54	–	–	(d) > (e) *

*p < 0.05, **p < 0.01, ***p < 0.001.

**TABLE 3:** Two-factor ANOVA and multiple comparisons for Leisure-oriented users.

**Personal space**	Interaction				Riding frequency		Before and during COVID-19
	***F*-value**	20.69***			3.85*		18.82***
	**Mean**	**SD**	**Mean**	**SD**	**Multiple comparisons**		**Multiple comparisons**
	**(d) Before**		**(e) During**		**(d) Before**	**(e) During**	
(a) Unchanged group	66.39	24.62	68.02	22.93	–	–	–
(b) Increased group	68.02	22.93	76.77	18.80	2* –	(b) > (a) *** (b) > (c)***	(e) > (d) ***
(c) Decreased group	66.10	23.62	64.17	24.33	–	–	–
**Time spent with others**	***F*-value**	26.34**			3.63*		**n.s.**
	**Mean**	**SD**	**Mean**	**SD**	**Multiple comparisons**		**Multiple comparisons**
	**(d) Before**		**(e) During**		**(d) Before**	**(e) During**	
(a) Unchanged group	59.45	26.15	59.26	26.68	–	–	–
(b) Increased group	63.15	26.72	68.06	23.35	2* –	(b) > (a)* (b) > (c)***	2* –
(c) Decreased group	60.34	26.25	55.44	26.98	–	–	–

*p < 0.05, **p < 0.01, ***p < 0.001. n.s., not significant.

In Tables 2, 3 and Figures 1–4, riding frequency is classified as (a) unchanged, (b) increased, and (c) decreased group. The mean score for the importance of personal space and time spent with others is divided between (d) before and (e) during COVID-19. The vertical axis of Figures 1–4 indicates the means scores for the importance of personal space and time spent with others, and the horizontal axis represents (d) before and (e) during COVID-19 as the timeline.

### Daily users

Table 2 and Figures 1, 2 show the results of the two-factor ANOVA and multiple comparisons for the daily users. The two-factor ANOVA results showed significant differences in the interaction effects for daily users in terms of both importance of personal space [*F*(2,867) = 28.85, *p* < 0.001] and time spent with others [*F*(2,867) = 18.43, *p* < 0.001].

The results of the simple main effect analysis for the importance of personal space showed significant differences in the motorcycle riding frequency condition [*F*(2,867) = 13.55, *p* < 0.001] and the pandemic condition [*F*(1,867) = 29.78, *p* < 0.001]. Multiple comparisons regarding riding frequency showed that the mean score of the increased group (*M* = 64.70, SD = 22.01) was significantly higher than that of the unchanged group (*M* = 56.22, SD = 23.91) before COVID-19, and that the mean score of the increased group (*M* = 74.36, SD = 19.71) was significantly higher than that of the unchanged (*M* = 57.27, SD = 23.69) and decreased groups (*M* = 56.68, SD = 25.95) during COVID-19.

Multiple comparisons between before and during COVID-19 indicated that the increased group’s mean score during COVID-19 (*M* = 74.36, SD = 19.71) was significantly higher than that before COVID-19 (*M* = 64.70, SD = 22.01) at a 0.001 significance level. The unchanged group indicated that the mean score during COVID-19 (*M* = 57.27, SD = 23.69) was significantly higher than that before COVID-19 (*M* = 56.11, SD = 23.91) at a 0.01 significance level. However, the difference in mean scores between these two groups might be small due to the minimal effect size (η2 = 0.007) (Cohen, 1988).

Regarding the importance of the simple main effect analysis for time spent with others, the results indicated significant differences in the motorcycle riding frequency condition [*F*(2,867) = 16.23, *p* < 0.001] and the pandemic condition [*F*(1,867) = 10.15, *p* < 0.001]. The increased group’s mean score before COVID-19 (*M* = 57.57, SD = 25.41) was significantly higher than that of the unchanged (*M* = 46.81, SD = 25.80) and decreased groups (*M* = 43.14, SD = 26.26). For during the COVID-19 condition, the increased group’s mean score before COVID-19 (*M* = 63.47, SD = 26.66) was also significantly higher than that of the unchanged (*M* = 46.94, SD = 25.95) and decreased groups (*M* = 46.46, SD = 26.54).

Multiple comparisons between before and during COVID-19 indicated that the mean score of the increased group during COVID-19 (*M* = 63.47, SD = 26.66) was significantly higher than that before COVID-19 (*M* = 57.57, SD = 25.41) at a 0.001 significance level. Concerning the decreased group, the mean scores before and during COVID-19 were not high and were significantly lower during COVID-19 (*M* = 41.46, SD = 26.54) than before COVID-19 (*M* = 43.14, SD = 26.26) at a 0.001 significance level. However, the difference in mean scores between these two groups could be small due to the minimal effect size (η2 = 0.005) (Cohen, 1988).

### Leisure-oriented users

Table 3 and Figures 3, 4 show the results of the two-factor ANOVA and multiple comparisons for the leisure-oriented users. The two-factor ANOVA demonstrated significant differences in the interaction effects for leisure-oriented users regarding the importance of personal space [*F*(2,887) = 20.69, *p* < 0.001] and time spent with others [*F*(2,887) = 26.34, *p* < 0.01]. Regarding personal space, the results of the simple main effect showed significant differences in the motorcycle riding frequency condition [*F*(2,887) = 3.85, *p* < 0.05] and pandemic condition [*F*(1,887) = 18.82, *p* < 0.001]. Significant differences in the mean score of the increased group (*M* = 76.77, SD = 18.80) were only evident during COVID-19. It was significantly higher than the mean scores of the unchanged (*M* = 68.02, SD = 22.93) and decreased groups (*M* = 64.17, SD = 24.33). Multiple comparisons concerning the increased group showed a significant difference between during COVID-19 (*M* = 76.77, SD = 18.80) and before COVID-19 (*M* = 68.02, SD = 22.93).

Regarding the simple main effect analysis for the importance of time spent with others, there were significant differences in the motorcycle riding frequency condition [*F*(2,887) = 3.36, *p* < 0.05]; however, no significant difference was found in the pandemic condition [*F*(1,887) = 0.996, *p* = 0.381, n.s.]. The simple main effect analysis for the increased group indicated that the mean score during COVID-19 (*M* = 68.06, SD = 23.35) was significantly higher than those of the unchanged (*M* = 59.26, SD = 26.68) and decreased groups (*M* = 55.44, SD = 26.98).

## Discussion

This study clarified whether riding a motorcycle serves as social interaction while practicing social distancing according to motorcyclists’ perceptions based on changes in the frequency of riding—unchanged, increased, and decreased—before and during the pandemic. The number of samples in the unchanged group was the highest among the three groups based on the frequency of motorcycle riding. Although respondents of the increased group were few, they perceive riding a motorcycle as necessary for keeping personal space and time spent with others, regardless of their purpose of possessing motorcycles, during the pandemic compared to before it.

Interestingly, while the importance of personal space and time spent with others were stipulated, there were no significant differences across the three groups of leisure-oriented users before COVID-19, and the importance of personal space for daily users was higher than in the other two groups. As mentioned in the literature review section, people maintain a distance from others that they find pleasant, and they feel uncomfortable when their personal space is invaded (Welsch et al., 2019). Motorcycles as a means of transportation in Asian countries are recognized as avoiding traffic jams, being more effective and efficient in using fuel, easier to buy compared to cars, and more economical than using public transportation (Yamamoto, 2009; Hanafi et al., 2019). Since one’s personal space is secured when riding a motorcycle, the survey results may reflect the attitudes of people who ride motorcycles daily. As a motorcycle is a substitute for public transportation for commuting in daily life (Huang et al., 2020; Scorrano and Danielis, 2021), riding a motorcycle could also be beneficial for the increased group to avoid crowded trains. In particular, as the mean score of the increased group during COVID-19 was 10 points higher, riding a motorcycle could be an essential tool for ensuring personal space during the pandemic.

Regarding the importance of time spent with others before COVID-19 for daily users, the mean scores of unchanged and decreased groups were lower than the mid-point of 50. However, the mean scores of the increased group before and during COVID-19 were significantly higher than the other two groups. Hence, daily users may usually ride a motorcycle as daily transportation as well as socializing, compared to the other two groups. Regarding the increased group, the mean scores of time spent with others significantly differed by approximately 20 points from the other two groups during COVID-19. More than 70% of daily users have a motorcycle with a displacement of less than 125cc. This suggests that riding a motorcycle, even one with a small engine, might lead to interaction with others among daily motorcycle users. This may have been more prevalent during the COVID-19 pandemic. The COVID-19 pandemic resulted in a prolonged period of limited social interaction, even within families (Sivan, 2020; Norbury, 2021). This was unprecedented, and the results of this study might be novel in determining the usefulness of motorcycle riding in terms of maintaining personal space and relationships with others.

Half of the leisure-oriented users possessed over 401cc displacement motorcycles, and about 70% of users have more than 251cc displacement motorcycles. According to mean scores for personal space of leisure-oriented users, they may have recognized the importance of personal space obtained from riding a motorcycle before COVID-19. A pandemic could have enhanced its importance in the increased group. In the increased group, although the importance of time spent with others also increased during COVID-19, the mean scores of the three groups were lower than the importance of personal space. Wellbeing related to riding a large-sized motorcycle for leisure-oriented users may have had a stronger connotation to free time and activities for mentally escaping from work, obligations, and other stressors, than for social interactions before COVID-19 (Zijlstra and Sonnentag, 2006; Liu et al., 2022). Its importance may have increased further during COVID-19 to prevent infection.

Motorcycle riding is a leisure activity, and the riding experience enhances the rider’s wellbeing (Kruger and Venter, 2020). The results of this study show that motorcycle riding is an effective way to spend time with others while maintaining personal space, a result that many people reaffirmed. Leisurely social interaction is a pivotal time and activity that leads to human wellbeing. Due to the pandemic, people have faced practicing social distancing continually and are compelled to restrict leisure activities. However, this study indicated that motorcycle riding could be a significant mean of practicing social distancing while spending time with others. Motorcycle riding may enable daily transportation and leisure-oriented users to practice social distancing while simultaneously spending time with companions and alleviating loneliness and isolation during the pandemic. When riding a motorcycle, riders are expected to pay sufficient attention to safety and improve their manners in order to control accidents and traffic congestion. But more than that, riding a motorcycle will provide us with various psychological benefits. A study on the psychological benefits of motorcycle riding would provide essential suggestions for post-pandemic lifestyle and leisure activities, ensuring personal space while spending time with others.

### Limitations and future research

This study compared the importance of motorcycle riding for personal space and time spent with others before and during COVID-19. For daily transportation and leisure activities, motorcycle riding as personal mobility enables users to practice social distancing and spend time with others, in terms of motorcyclists who increased their motorcycle riding frequency. Our findings might contribute to human wellbeing through two-wheel personal mobility. However, this study has some limitations.

First, although this study indicated the importance of personal space and time spent with others by comparing before and during COVID-19, the specific reasons for this importance are still unclear. The psychological and behavioral aspects of the riders could undeniably be affected by several factors, such as particular occasions or legal regulations. Research clarifying the reasons and triggers for increasing motorcyclists’ personal space and time may be necessary while considering the factors influencing their motorcycle riding.

Second, the terms for personal space and time spent in this study relied on respondents’ interpretations. Occasions involving social interactions may have varied. This study was not limited to either riding alone or with others. Additionally, social interaction could imply riding with two people and interacting with other motorcyclists. Some motorcyclists form brand communities of particular motorcycle manufacturers (Marzocchi et al., 2013). However, this study did not discuss the types of interactions. Thus, future studies are required to understand the specific social interactions and the meaning of personal space induced by riding motorcycles. We did not consider information regarding passengers in the present study. Riders can be divided into two categories: those who ride alone and those who go touring in groups. Since about 80% of riders in Japan ride solo, especially men, we assumed that most participants in this study ride solo (Japan Automobile Manufacturers Association, Inc [JAMA], 2022). In future studies, it will be necessary to examine the effects of difference in terms of passenger on personal space and social interaction.

Finally, as Japan did not implement lockdowns for behavior restrictions, the riding frequency was measured according to personal perception. However, we did not include the respondents’ relationship with motorcycles, such as their riding history and experiences or how long they have owned motorcycles. Depending on their psychological and behavioral relationships with motorcycles, the importance of motorcycles during COVID-19 may differ. Research is consequently required to examine the perceived importance of riding a motorcycle for motorcycle users before and after COVID-19 to secure personal space and time, as well as activities for social interactions. Additionally, the importance and kind of values remain unclear for motorcyclists. Research that illustrates motorcycles as personal mobility and how they contribute to the wellbeing of humans could thus be essential.

## Data availability statement

The original contributions presented in this study are included in the article/supplementary material, further inquiries can be directed to the corresponding author.

## Author contributions

YW conceived and designed the research and wrote the first draft of the manuscript. YB wrote sections of the manuscript. MI supervised the work. All authors have read and approved the manuscript.
